# 

**Published:** 1877-10

**Authors:** 


OCTOBER, 1877.

### THE AMERICAN JOURNAL OF DENTAL SCIENCE.

EDITED BY

F. J. S. GORGAS, M. D, D. D. S.,

AND

JAMES B. HODGKIN, D. D. S.

VOL. 11.—THIRD SERIES.—No. 6.

BALTIMORE:

SNOWDEN & COWMAN.

LONDON:

TRUBNER & CO., 60 PATERNOSTER ROW.

1877.

PAYABLE IN ADVANCE.

PUBLISHED MONTHLY.

$2.50 PER ANNUM.

